# Occupations of Life

**Published:** 1859-08

**Authors:** 


### OCCUPATIONS OF LIFE.

When a youth is about determining what he shall follow for a living, the first rule is to select the employment which he likes best; one which he can follow *con amore*, that is, with the most satisfaction to his inclinations, tastes or desires; always pre-supposing, that it is not merely an allowable calling, but one that is useful and honorable.

The second inquiry should be, will health admit of it? Sickly, or even merely feeble persons should not think for a moment, of any indoor occupation. It is worse than suicidal, because, besides the risk of destroying their own lives, there are chances of this being done not soon enough to prevent the introduction of a diseased progeny, to be life long miserables themselves, and to be a burden to others. Of the in-door occupations, some of the most trying to the human constitution are working in cotton, hemp, paints, dyeing furs, tobacco, lucifer matches, manufacturer's trimmings, and the like, involving the filling of the air with minute particles.

Blondes, that is, persons with light hair, fair skin, and blue eyes; as also those having sandy or reddish hair, should, by all means, select some active, out-door vocation.

Brunettes, persons having a dark skin, indicating the bilious temperament, accompanied usually with black hair, and dark eyes, should select a calling which, whether indoor or out, will require them to be on their feet, moving about nearly all the time, in order to "work off" the constantly accumulating bile.

The mixed temperaments can best bear sedentary in-door occupation; such as a combination of the bilious and nervous. Spare persons, not having much flesh, but enough of the nervous and sanguine temperament to give them a wiriness of constitution, these can bear in-door occupations best; their activity arising from the nervous temperament keeping them in motion, (the tongue any how, if women,) while their hopefulness, arising from the sanguine temperament, keeps up their spirits, which is an element as essential to success, as it is to health.

But of all human occupations which do not render a man amenable to the laws of his country, the most universally and invariably destructive to the health of the body, as well as that of the mind and heart, and yet coveted by many, although it is the hardest work in the world, is that of having nothing to do.

